# Near infrared multiphoton-induced generation and detection of hydroxyl radicals in a biochemical system

**DOI:** 10.1016/j.abb.2007.04.026

**Published:** 2007-08-15

**Authors:** Stanley W. Botchway, Ana G. Crisostomo, Anthony W. Parker, Roger H. Bisby

**Affiliations:** a Biomedical Sciences Research Institute, University of Salford, Salford M5 4WT, UK; b Central Laser Facility, STFC Rutherford Appleton Laboratory, Chilton, Didcot, Oxfordshire OX11 0QX, UK

**Keywords:** Radical, Hydroxyl, Scavenging, Multiphoton, Microscopy, Mercaptopyridine-*N-*oxide, PhotoFenton, Spectroscopy, Laser, Femtosecond

## Abstract

Solutions of tryptophan and *N-*hydroxypyridine-2-thione (mercaptopyridine-*N-*oxide, MPNO) were irradiated at 335 nm. Formation of 5-hydroxytryptophan was inferred from increased fluorescence at 334 nm on excitation at 315 nm, conditions chosen for selective detection of 5-hydroxytryptophan. Such experiments are complicated by overlapping absorption spectra in the region of 300–350 nm. Similar solutions were exposed to multiphoton excitation at 750 nm using 180 fs pulses from a titanium:sapphire laser. In solutions containing both tryptophan and MPNO strong emission at 500 nm was observed that was absent in solutions containing either MPNO or tryptophan only. This emission is ascribed to the characteristic fluorescence (‘hyperluminescence’) from 5-hydroxyindoles resulting from multiphoton photochemistry. The conclusion that MPNO generates hydroxyl radicals by 2-photon activation at 750 nm is confirmed by the scavenging effects of ethanol and kinetic analysis of the results. This method has potential applications in intracellular induction of oxidative stress using multiphoton near-infrared illumination, a technology that is gaining momentum as a research tool.

The detection of reactive oxygen species (ROS),^1^ such as hydroxyl radicals, in biological systems is challenging due to their high reactivity and extremely short lifetimes that are typically sub-microsecond [1,2]. A frequent approach is to use a scavenger which leads to distinctive products: examples include salicylate which is hydroxylated to a characteristic ratio of isomeric products [3,4] and dimethylsulfoxide which is oxidised to methanesulfinate by hydroxyl radical [5,6]. Spin traps have been extensively used for such purposes [7,8] and this permits indirect imaging of radical species in tissues such as skin [9]. Luminescent probes have also been used to detect oxidative stress in cellular systems, and examples include 2′,7′-dichlorodihydrofluorescein and dihydrorhodamine which are oxidised to their fluorescent products [10], although there is some concern as to their specificity regarding the type of ROS (hydroxyl, superoxide etc) detected. Further consequences of ROS activity within cells are important and secondary biological products stemming from radical chemistry can also be investigated using imaging techniques, for example lipid peroxidation [11,12]. At the more chemically specific level tryptophan is one of the most reactive amino acids and is subject to attack by electrophilic reagents such as the hydroxyl radical, reacting with a near diffusion controlled rate constant [13]. In this respect there have been numerous studies showing how tryptophan is degraded in proteins exposed to hydroxyl radicals and use of this is made in the ‘footprinting’ method for probing protein surfaces [14,15]. Tryptophan is also a target for reaction of reactive nitrogen species such as peroxynitrite [16].

There is currently much interest in oxidative stress in the cellular environment in relation to biochemical responses such as apoptosis and DNA repair. Continuous levels of oxidative stress may be induced by peroxyl radical generators such as the azo-initiators or through Fenton reagents. However, the opportunity for spatial and temporal control of oxidative stress using photo-activated precursors is now possible. Hydroxyl radicals may be generated by ultraviolet excitation of compounds such as pyridine-*N-*oxides, often referred to as “photoFenton” reagents [17–19]. The generation of hydroxyl radicals from such reagents has been identified by trapping with coumarin-carboxylic acid and the consequent formation of the highly fluorescent 7-hydroxycoumarin carboxylate [20]. A potential problem with one-photon excitation of photoFenton reagents such as 2-hydroxypyridine-*N-*oxide (HPNO) and 2-mercaptopyridine-*N-*oxide (MPNO) are their short wavelength (UV) absorption maxima at ∼310 and ∼330 nm respectively at physiological pH (see Fig. 2). Such wavelengths are absorbed by several biochemical chromphores and so have limited penetration in tissues. The use of multiphoton excitation in the near infrared (ca 700–900 nm) with a femtosecond titanium-sapphire (Ti:Saph) laser has the potential to circumvent these problems and also allows pseudo-confocal three dimensional microscopic imaging of cellular systems due to the femtolitre volume at the focus of the laser beam [21–23]. Although cross-sections for 2- and 3-photon absorption are many orders of magnitude less than for the corresponding one-photon process, the high peak power within a ca 100 fs laser pulse may be used to induce photochemistry such as uncaging [24] and generation of reactive oxygen species from the triplet states of dyes [23,25]. Focusing these extreme peak laser powers (MW to GW) may cause dielectric breakdown in the solvent and Nishimura and Kinjo [26] have reported the formation of a green emission in solutions of tryptophan exposed to femtosecond laser pulses with peak power densities up to 1.2 × 10^12^ W cm^−2^. Shear et al. [27,28] were the first to report an unusual green emission (‘hyperluminescence’) from serotonin on multiphoton near infrared excitation. This was shown to be due to the generation of a photochemical product in a 4-photon event at 830 nm that is subsequently excited by a further two photons to generate the green fluorescence. Although such hyperluminescence has been further characterised [29,30], the nature of the emitting species remains unknown although it appears to be relatively specific for 5-hydroxyindoles. Laser flash photolysis has identified a triplet state. Together with neutral and cation radicals similarly identified, these are likely to be involved as precursors of the emitting state [31]. The observation of green luminescence from tryptophan solutions exposed to high laser powers [26] suggests the formation of hydroxyl radicals from laser-induced breakdown of water and subsequent formation and excitation of 5-hydroxytryptophan. We have now explored the possibility of hydroxyl radical generation by 2-photon excitation of photoFenton agents and its detection from reaction with tryptophan.

## Materials and methods

All chemicals were obtained from Sigma–Aldrich and used as received. Solutions were prepared in triply filtered Milli-Q purified water (total organic content <2 ppm) and buffered to pH 7.3 with phosphate. Fluorescence spectra were measured in a Spex Fluoromax spectrofluorimeter equipped with a stirred cell compartment. Absorption spectra were measured with a Perkin Elmer Lambda 35 UV–vis spectrophotometer. Non-linear curve fitting used the Grafit software package.

**Fig. 1:**
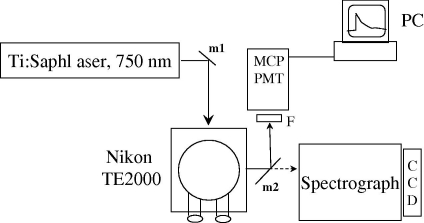
Schematic of the apparatus used for the experiments. The Ti:S laser produces 180 fs pulses at 75 MHz with a total average power of up to ∼100 mW at the sample. The power is adjusted by a variable neutral density filter (m1) before entering the epifluorescence microscope (Nikon TE2000). Emission is detected via a movable mirror (m2) either spectrally using a spectrograph–CCD combination or in time-resolved mode using an interference filter and microchannel plate photomultiplier (MC-PMT) linked to a time-resolved single photon counting card in the PC.

The apparatus for multiphoton experiments (Fig. 1) used the output of a Ti:Saph (Mira, Coherent Ltd) laser operating at 750 nm and producing up to 1.2 W in 180 fs pulses at 75 MHz. Power output was measured with a Molectron 500D power meter. About 8% of laser power was transmitted to the sample and Figures and Legends indicate the average power determined at the sample position. The laser beam was coupled via a mirror and variable density filter for power adjustment (m1) through a telescope to a Nikon TE2000 fluorescence microscope with a 60× water immersion objective. An output port of the microscope was optically coupled via a movable mirror (m2) to a spectrometer (Acton Spectrodrive 275) and CCD (Andor iDus, model DU440) camera for measurement of emission spectra, or to a Hamamatsu R3809U microchannel plate photon-counting photomultiplier tube linked to Becker and Hickl SPC830 PC card and software for fluorescence lifetime measurements. For measurement of spectra, a 6640IK dichroic filter (Comar) was used in combination with a CuSO_4_ solution filter placed in front of the spectrograph, whilst for lifetime measurements of 500 nm emission, BG39 and 500 ± 10 nm interference filters (F) were placed in front of the photomultiplier. Sample solutions were placed as drops on a No. 1 glass cover slip (35 mm dia, 190 μm thick) clamped between two stainless steel plates so as to be rigidly located on the microscope sample stage and allowing samples to be changed without altering the precise focus within the solution required to image the confocal volume onto the detectors. Spectra measured using the CCD array were integrated for between 5 and 60 s and are shown after subtraction of a corresponding spectrum from water alone which also resulted in subtraction of the detector dark/readout count. The apparent structure in some of the spectra shown arises from the transmission curves of the interference filters used.

## Results and discussion

### UV excitation

**Fig. 2:**
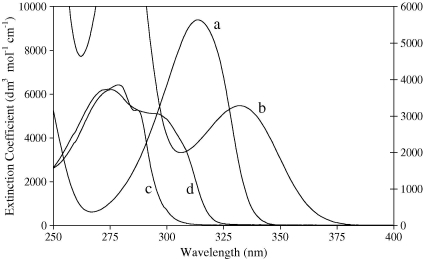
Absorption spectra of HPNO (a) and MPNO (b) (right-hand scale) shown with those of Trp (c) and 5-OHTrp (d) (left-hand scale). All spectra were measured in phosphate buffered saline (pH 7.3).

Both MNPO and HPNO have been shown to be “photoFenton” reagents, liberating free hydroxyl radicals on illumination with quantum yields of ca. 0.11–0.28 [19]. Fig. 2 shows the absorption spectra of MPNO, *λ*_max_ 332 nm, and HPNO, *λ*_max_ 313 nm, which overlap with the fluorescence spectra of both Trp (*λ*_max_ 350 nm) and 5-hydroxytryptophan (5-OHTrp, *λ*_max_ 334 nm). Also shown in Fig. 2 are the absorption spectra of Trp and 5-OHTrp, illustrating the opportunity to selectively excite 5-OHTrp at 315 nm in mixtures of the two solutes. Despite this spectral congestion it is possible in the sample compartment of a fluorimeter to sequentially generate hydroxyl radicals in solution by a period of illumination at 335 nm, see reaction (1), followed by determination of 5-OHTrp formation (reaction (2)) through measurement of fluorescence at 334 nm on excitation at 315 nm.

(1) $$\text{MPNO}\overset{\mathrm{hv}}{\underset{355\text{nm}}{\to }}{}^{}\text{OH}+\text{product}$$

(2) ·OH+Trp→5-OHTrp

(3) ·OH+MPNO→products

**Fig. 3:**
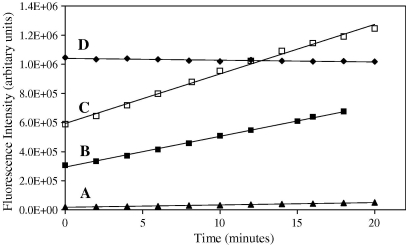
Detection of 5-hydroxytryptophan fluorescence on excitation of solutions of MPNO and Trp at 315 nm. The solutions were irradiated at 335 nm (slits 30 nm bandwidth) in a stirred 1 cm quartz cuvette in the sample compartment of a Spex Fluoromax fluorimeter for 2 min periods. Following each irradiation period, fluorescence was measured at 334 nm on excitation at 315 nm (5 nm slits). Solutions were air-saturated in phosphate buffer (0.05 mol dm^−3^, pH 7.3). Line A—MPNO (200 μmol^−3^) only; line B—trp (250 μmol dm^−3^) and MPNO (200 μmol^−3^); line C—trp (500 μmol dm^−3^) and MPNO (200 μmol dm^−3^); line D—trp (250 μmol dm^−3^) only.

Fig. 3 shows the results from such an experiment. Only in the presence of both MPNO (0.2 mmol dm^−3^) and Trp (lines B (0.25 mmol dm^−3^) and C (0.5 mmol dm^−3^)) is a significant increase in fluorescence observed. Despite the wavelengths chosen for selective excitation of 5-OHTrp at 315 nm, considerable Trp fluorescence is observed under the conditions of the experiment (line D). A small non-interfering amount of fluorescence is also observed from MPNO alone (line A). A considerable inner filter effect reduces the intensities of 334 nm fluorescence due to MPNO absorbance (see Fig. 2) when comparing the Trp only solution (line D) and the mixtures of Trp and MPNO (lines B and C). Increasing the Trp concentration from 0.25 mmol dm^−3^ (line B) to 0.5 mmol dm^−3^ (line C) increases the rate of 5-OHTrp formation by a factor of 1.62, since reaction (2) now competes more effectively for hydroxyl radicals than reaction (3). The difficulties of using excitation and emission due to overlapping absorption curves of the solutes in the UV region are well illustrated by the data shown in Figs. 2 and 3.

### Multiphoton near-infrared excitation

Using multiphoton excitation with 180 fs pulses in the near-infrared for activation of this photochemistry has a number of advantages, including simultaneous photoexcitation of both photoFenton agent, generating hydroxyl radical, and 5-OHTrp, permitting generation of the specific green (‘hyperluminescence’) emission [27–29] from 5-OHTrp. In multiphoton processes there is no competing absorption by solutes as there is in the one-photon case. Consequently, the inner filter effect at 750 nm is almost completely absent.

**Fig. 4:**
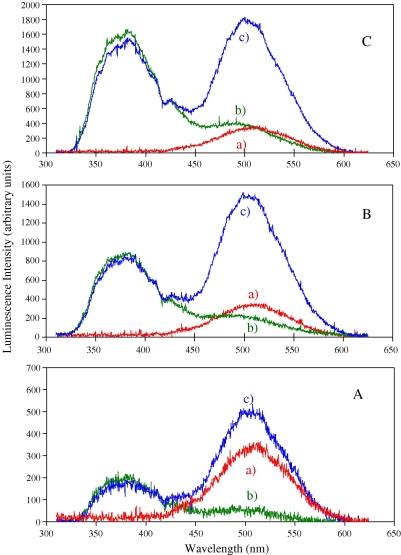
Emission spectra measured using a CCD array on multiphoton illumination (750 nm, 180 fs pulses at 75 MHz, average power 35 mW) of solutions of Trp and MPNO (2 mmol dm^−3^) in phosphate buffer (pH 7.3, 50 mmol dm^−3^). Each figure shows spectra from MPNO alone (curve a)), Trp alone (curve b)) and Trp and MPNO together (curve c)) at Trp concentrations of 0.45 mmol dm^−3^ (A), 1.8 mmol dm^−3^ (B) and 3.6 mmol dm^−3^ (C). Accumulation time 200 s.

Fig. 4 shows the luminescence spectra obtained on multiphoton excitation at 750 nm of air-saturated solutions of Trp and MPNO. The spectra were obtained with the microscope coupled to a spectrograph and CCD detector configured to reject wavelengths below 340 nm. The spectra are uncorrected for relative differences in transmission across the spectral window in the optical system, due mainly to the filters and microscope objective, and for the spectral response of the detector. The solutions contain increasing concentrations of Trp (0.45–3.6 mmol dm^−3^) and a fixed concentration of MPNO (2 mmol dm^−3^). The solutions containing only Trp reveal the red edge of the normal UV fluorescence (*λ*_max_ 350 nm) in addition to a weak peak at 500 nm (vide infra). Solutions of MPNO also show a weak emission with *λ*_max_ ca 505–510 nm. In solutions containing both Trp and MPNO a stronger emission at 500 nm is observed and ascribed to hyperluminescence from 5-OHTrp, formed by photochemical multiphoton formation of hydroxyl radicals from MPNO (reaction (1)) followed by their reaction with Trp to form 5-OHTrp (reaction (2)). In contrast to the results of the one-photon excitation in Fig. 3 that show an increase in 5-OHTrp fluorescence with time, the intensities measured from the multiphoton experiment (Fig. 4) are time-independent for over an hour of continuous irradiation from the point of initial exposure of the solution to the laser beam. We suggest that this steady state is due to a rapid equilibrium being established between creation of intermediates within the femtolitre confocal volume in which multiphoton events take place at the laser beam focus and diffusion of photochemical products out of the confocal volume into bulk solution where they are no longer detected. Calibration with solutions of 5-OHTrp show that for the solution in Fig. 4C containing MPNO and Trp (3.6 mmol dm^−3^) the observed intensity corresponds to the formation of about 160 μmol dm^−3^ of 5-OHTrp by multiphoton-induced reactions (1) and (2) within the confocal volume.

Similar experiments were undertaken with the related photoFenton reagent, HPNO, but failed to show any sensitised emission at 500 nm. Inspection of the absorption spectra in Fig. 2 shows that absorption by MPNO (spectrum b) extends to 375 nm, corresponding to 2-photon excitation at 750 nm. In contrast HPNO absorption (spectrum a) has a long wavelength limit at ∼350 nm, and it appears that there is insufficient cross section for 2-photon excitation at 750 nm.

### Time-resolved fluorescence measurements

**Fig. 5:**
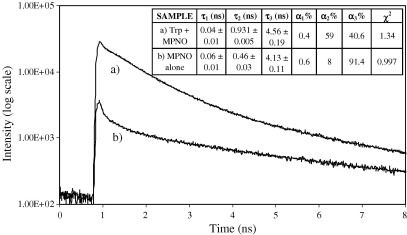
Nanosecond time-resolved fluorescence decay decays recorded from multiphoton excitation at 750 nm of solutions of (a) tryptophan (2 mmol dm^−3^) plus MPNO (2 mmol dm^−3^) and (b) MPNO (2 mmol dm^−3^). Emission was detected through a 500 nm interference filter.

Further confirmation that the enhanced 500 nm emission is due to hyperluminescence from 5-OHTrp is obtained from the un-deconvoluted time-resolved fluorescence decays obtained with 750 nm excitation shown in Fig. 5 together with triple exponential analyses. Analysis of each decay shows that they contain a fast (ca 50 ps) component due to scattering of the laser excitation pulse within the microscope optics with a longer time constant than that of the laser pulse due to the system (photomultiplier plus electronics) response function. For the solution of MPNO there are further components of 460 ps and 4.13 ns. In contrast, the solution containing the mixture of Trp and MPNO and showing the strong green emission has a predominant decay time of 0.93 ns and a further component with a lifetime of 4.56 ns. The dominant fluorescence component with *τ* 0.93 ns clearly corresponds to that measured for multiphoton induced hyperluminescence from solutions of 5-OHTrp (*τ* 0.91 ns [29]) and confirms the green emission as arising from 5-OHTrp.

### Emission from multiphoton excitation of solutions of tryptophan and MPNO

**Fig. 6:**
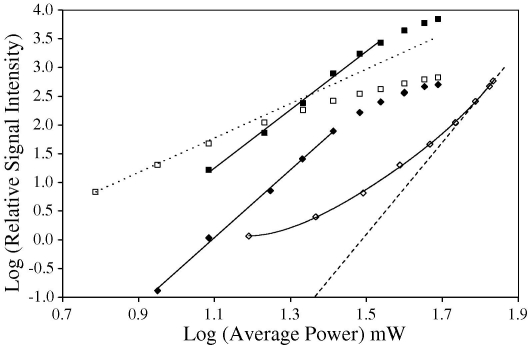
Log–log plot showing the effect of average laser power at 750 nm on emission intensity at:- 510 nm in solutions containing MPNO (2 mmol dm^−3^) only (♦, solid line slope of 5.9); at 500 nm (■, solid line slope of 5.1) and at 380 nm (□, dotted line slope of 3) in solutions of Trp (4 mmol dm^−3^) and MPNO (2 mmol dm^−3^); and at 500 nm in solutions of Trp (2.5 mmol dm^−3^) only (◇, dashed line slope of 8).

Fig. 6 shows the effect of laser power on intensities of both the UV fluorescence and hyperluminescence from a solution of tryptophan and MPNO. The normal UV fluorescence originates from a 3-photon excitation at 750 nm. The dotted curve in the log–log plot has a slope of 3 and confirms the 3-photon mechanism at low powers. However, above an average power of 15 mW saturation occurs and the intensity falls below that predicted. The green emission from the same solution was measured to have a slope of 5.07 ± 0.16 in the log–log plot indicating an overall 5-photon process at 750 nm, compared with a 6-photon excitation reported at 830 nm [27]. The power dependence of the weaker emission at 510 nm from solutions of MPNO was found to have a slope of 5.90 ± 0.16 in the log–log plot. All of these samples showed a linear response in the log–log plot at lower laser powers but saturated at higher powers, indicating photochemical degradation of ground states and intermediates at such high powers densities (>5 × 10^11^ W cm^−2^). At average laser powers greater than 20 mW significant green emission was also observed from solutions of tryptophan alone and increases steeply in intensity with increasing power. The non-linear curve in the log–log plot shows an increasing absorption cross section for this process above a threshold value and a limiting slope at the highest powers used in our experiments of about 8. These results are rather similar to those obtained by Nishimura and Kinjo [26] using 830 nm multiphoton excitation. It is believed that at such high powers dielectric breakdown in water induces the formation of radical species, including hydroxyl radical, which then react with tryptophan to give the 5-hydroxytryptophan signal. Our experiments with Trp and MPNO shown in Fig. 4 therefore use a laser power which is a compromise between that sufficiently high to produce a reasonable hyperluminescence signal in an overall 5-photon process but not so high as to induce significant radical formation by solvent breakdown.

### Effect of Trp concentration

**Fig. 7:**
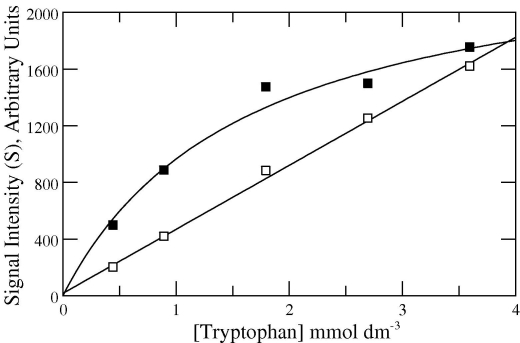
Effect of Trp concentration on the intensities of fluorescence at 380 nm (□) and 500 nm (■) measured on 750 nm multiphoton excitation of solutions containing MPNO (2 mmol dm^−3^) in phosphate buffer. The curve shown for the 500 nm emission is that obtained by non-linear fitting of the data to Eq. (4).

Compared with the corresponding solutions of Trp alone (Fig. 4, curves b), the intensities at 380 nm of the mixed solutions (Trp + MPNO, curves c) are almost unchanged and indicate essentially no interference between normal fluorescence and formation of 5-OHTrp and the 500 nm emission. Fig. 7 shows that the fluorescence intensities at 380 nm generated through multiphoton excitation of solutions containing both Trp and MPNO increase linearly with Trp concentration, as would be expected for solutions with low absorbance at the excitation wavelength. In contrast the plots of luminescence intensity at 500 nm are curved and approach a limiting value as Trp concentration is increased. Since the MPNO concentration is fixed, the amount of hydroxyl radical generated is expected to remain constant, and the results reflect the competition between scavenging of ^•^OH by Trp and MPNO, reactions (2) and (3) (with second order rate constants *k*_2_ and *k*_3_ respectively). The luminescent signal at 500 nm (*S*) may be related to its maximum value (*S*_max_) by:-

(4) $$\frac{S}{S_{\max}}=\frac{k_{2}[\text{Trp}]}{k_{2}[\text{Trp}]+k_{3}[\text{MPNO}]}$$

This kinetic analysis ignores bimolecular recombination of hydroxyl radicals, since their steady state concentration is expected to be at least 3 orders of magnitude less than those of Trp and MPNO. Non-linear least squares fitting to Eq. (4) of the curve for the 500 nm signal in Fig. 7 gives *k*_3_/*k*_2_ = 0.82 ± 0.23. Taking the second order rate constant for reaction of ^•^OH with tryptophan, *k*_3_, as 1.4 × 10^10^ dm^3^ mol^−1^ s^−1^ [13] gives a value for *k*_2_ of (1.1 ± 0.3) × 10^10^ dm^3^ mol^−1^ s^−1^. Aveline et al. [18] quote a value of *k*_2_ measured by pulse radiolysis of 9 × 10^9^ dm^3^ mol^−1^ s^−1^, whilst the related compound HPNO reacts with ^•^OH with a second order rate constant of 2 × 10^10^ dm^3^ mol^−1^ s^−1^ [32]. Applying this kinetic model to the data therefore produces a reasonable fit to the known kinetics of the system and confirms our interpretation of the multiphoton chemistry.

### Scavenging by ethanol

**Fig. 8:**
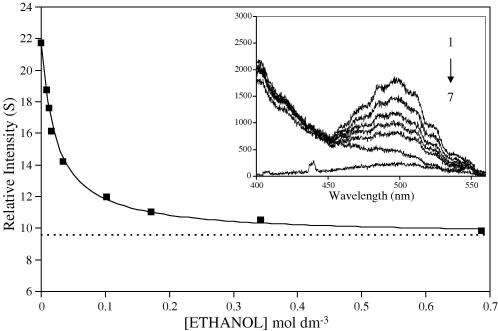
Effect of ethanol addition on the relative intensity (*S*) of hyperluminescence at 500 nm from solutions of tryptophan (4 mmol dm^−3^) and MPNO (2 mmol dm^−3^). The solid line shows the data fitted to Eq. (5). *Inset.* Luminescence spectra from solutions of Trp and MPNO containing ethanol at concentrations of 0 (curve 1), 12.9 (curve 2), 34.3 (curve 3), 103.0 (curve 4) and 687 mmol dm^−3^ (curve 5). Also shown are spectra from solutions containing only Trp (curve 6) and MPNO (curve 7). Apparent structure in these curves is due to transmission characteristics of the interference filters as noted in Materials and methods.

Ethanol is an effective scavenger of hydroxyl radical with a second order rate constant (*k*_e_) of 1.9 × 10^9^ dm^3^ mol^−1^ s^−1^ [13], producing carbon-centred radicals which react with oxygen to form peroxyl radicals with modest reactivity. It is not anticipated that either the radicals or chemical products from ethanol will react with tryptophan to give 5-OHTrp. Thus the addition of ethanol to solutions of Trp and MPNO are expected to reduce the yield of luminescence at 500 nm and results from experiments demonstrating this are shown in Fig. 8. At high concentrations of ethanol over 90% of the additional signal in the mixed solution at 500 nm (the difference between curves (c and b) in Fig. 4C) is removed. This observation is good evidence that photolysis of MPNO produces a reactive radical such hydroxyl, which is then involved in production of 5-OHTrp and hyperluminescence on multiphoton irradiation. The validity of this is supported by kinetic analysis of the data shown in Fig. 8. The intensity of the emission at 500 nm (*S*) is proportional to the fraction of the hydroxyl radicals scavenged by Trp in competition with both MPNO and ethanol, plus the background signal (*B*) observed from the individual solutions of Trp and MPNO,

(5) $$S=\frac{k_{2}[\text{Trp}]}{k_{2}[\text{Trp}]+k_{3}[\text{MPNO}]+k_{\text{e}}[\text{EtOH}]}+B$$

The best fit to the data to Eq. (5) is shown by the solid curve in Fig. 8 taking *k*_e_ to be 3.5 × 10^9^ dm^3^ mol^−1^ s^−1^, which is in reasonable agreement with the actual value [13].

Overall the results described here show that multiphoton excitation of MPNO at 750 nm generates the hydroxyl radical and this may then be scavenged by tryptophan to yield 5-hydroxytryptophan that is subsequently excited in a 5-photon process similar to that previously described [27] to produce the characteristic green emission. It is also possible that hydroxyl radicals react with tryptophan to give another radical product (such as the 5-indoxyl radical) which might be excited to produce the fluorescence. However, the evidence points towards the formation of 5-hydroxytryptophan as this can be detected in the one-photon experiment from UV-excited fluorescence and the spectroscopic properties of the green emission (wavelength, fluorescence lifetime) are essentially identical to those determined previously for multiphoton excitation of 5-OHTrp. Furthermore, the relatively high concentration of 5-OHTrp within the confocal volume indicated by the relative intensity of the green emission suggests that 5-OHTrp accumulates between successive sub-picosecond pulses of the Ti-Sapphire laser.

## Conclusions

The results show that multiphoton near-infrared excitation of the photoFenton reagent MPNO is capable of generating significant yields of hydroxyl radical within the confocal volume at the laser focus. Simultaneous detection of hydroxyl radicals was enabled by adding tryptophan as a hydroxyl radical scavenger producing 5-OHTrp which was re-excited to form a species emitting at 500 nm. This may have the potential to detect hydroxyl radical generation in more complex biochemical and biological systems. In comparison with the normal one-photon excitation in the UV, the near-infrared excitation has several advantages. These include the lack of competing absorption by the solutes due to their lower cross sections at 750 nm, the simultaneous excitation of the probe, and the potential to be applied to cellular systems for the study of hydroxyl radical induced stress. In the latter context, oxidative stress could be induced at a specific intracellular locus as is currently being explored by direct photodamage of DNA [33,34] and resulting effects imaged in the scanning confocal microscope. The results demonstrate the feasibility for developing further the use of multiphoton activated ROS for studying oxidative stress at the chemically specific level within living cells.

## Footnotes

1 *Abbreviations used:* ROS, reactive oxygen species; HPNO, 2-hydroxypyridine-*N-*oxide; MPNO, 2-mercaptopyridine-*N-*oxide; 5-OHTrp, 5-hydroxytryptophan.
